# Development of a Method Suitable for High-Throughput Screening to Measure Antioxidant Activity in a Linoleic Acid Emulsion

**DOI:** 10.3390/antiox8090366

**Published:** 2019-09-02

**Authors:** Md Ahsan Ghani, Celia Barril, Danny R. Bedgood, Paul D. Prenzler

**Affiliations:** 1 School of Agricultural and Wine Sciences, Faculty of Science, Charles Sturt University, Wagga Wagga 2650, Australia; 2 Graham Centre for Agricultural Innovation, Charles Sturt University, Wagga Wagga 2650, Australia

**Keywords:** thiobarbituric acid reactive substances, ferric thiocyanate, high-throughput screening, bioactivity, plant extracts, method validation, lipid oxidation, peroxidation

## Abstract

An improved system for measuring antioxidant activity via thiobarbituric acid reactive substances and ferric thiocyanate assays is reported, on the basis of oxidation of a linoleic acid (LA) emulsion. Oxidation times were reduced from 20 h to 5 h by increasing the reaction temperature from 37 °C to 50 °C and with an acceptable precision of <10% coefficient of variation (CV). Antioxidants varying in polarity and chemical class—250 µM Trolox, quercetin, ascorbic acid and gallic acid—were used for method optimisation. Further reductions in reaction time were investigated through the addition of catalysts, oxygen initiators or increasing temperature to 60 °C; however, antioxidant activity varied from that established at 37 °C and 20 h reaction time—the method validation conditions. Further validation of the method was achieved with catechin, epicatechin, caffeic acid and α-tocopherol, with results at 50 °C and 5 h comparable to those at 37 °C and 20 h. The improved assay has the potential to rapidly screen antioxidants of various polarities, thus making it useful in studies where large numbers of plant extracts require testing. Furthermore, as this assay involves protection of a lipid, the assay is likely to provide complementary information to well-established tests, such as the 2,2-diphenyl-1-picrylhydrazyl (DPPH) assay.

## 1. Introduction

Lipid oxidation is problematic in the food industry as it leads to rancidity [1], and is more broadly detrimental to human health as it is implicated in such diseases as atherosclerosis, cancer and arthritis [2]. Polyunsaturated fatty acids (PUFAs), such as linoleic acid (LA), are very susceptible to oxidation in the presence of heat, light or trace amounts of metal ions. This oxidation produces harmful primary oxidation products such as peroxides, which breakdown to numerous toxic secondary oxidation products, such as aldehydes, carbonyl compounds, conjugated dienes and furans. Some of these, including malondialdehyde (MDA), react with thiobarbituric acid (TBA) to give pink/red coloured adducts, which are measured in the thiobarbituric acid reactive substances (TBARS) test. On the other hand, peroxides may be measured by the ferric thiocyanate (FTC) assay. The latter assay is commonly used for dairy products such as milk or fats [3,4] while the TBARS assay is used for meat products [5] and also in human physiology [6].

Antioxidants are compounds that can inhibit the formation of oxidation products when present at a much lower concentration compared to an oxidisable substrate. An antioxidant may function in several ways: neutralising free radicals formed in oxidation, chelating metal ions or scavenging oxygen and reactive oxygen species (ROS). There is currently much interest in screening the antioxidant activity of plant extracts (e.g., traditional medicinal plants) or foods to investigate potential medicinal properties, or alternatively to understand the link between diet and health. Free radical scavenging assays, such as 2,2′-azino-bis (3-ethylbenzothiazoline-6-sulphonic acid) (ABTS) or 2,2-diphenyl-1-picrylhydrazyl (DPPH) assays, are commonly used to measure antioxidant activity because of their simplicity and capability in high-throughput screening. These assays provide some information as to the mechanism of antioxidant activity (e.g., electron transfer, hydrogen atom transfer, etc.); however, they lack a substrate, and therefore do not provide a measure of the protection that the antioxidant affords an oxidisable molecule.

On the other hand, both FTC and TBARS assays can be used to measure the protection of a lipid substrate in the presence of an antioxidant. A variety of lipid substrates have been used in screening studies on plant extracts, such as LA on water [7] or in emulsion (oil-in-water) [8], liposome [9] or rat organs (such as the liver, heart, etc.) [10]. However, for comparative purposes across different research groups, some substrates may not be reproducible in terms of their lipid content (e.g., animal models) and even in liposomes. Buenger et al. [9] reported high intra- and inter-laboratory variability in the TBARS assay, especially with tocopherol as the antioxidant. As it is available in high (>99%) purity, LA may appear to be an ideal substrate for screening studies. However, in a recent report, we have demonstrated problems with LA when used as a separate phase on top of water [11]. Conversely, when LA was used in an emulsion system, many groups have reported good precision in both the TBARS and FTC tests [12,13,14]. Thus, because of its low cost, relatively simple oxidation reactions (relative to higher PUFA), and its ready availability in high purity, LA appears to hold much promise as a substrate for screening antioxidant activity when used in an emulsion.

Using an LA emulsion, there are a number of reports where oxidation was conducted at 37 °C, with FTC [8], TBARS [15] or both assays [16]. With these studies, the choice of 37 °C was due to the fact that it is a physiologically relevant temperature, and it is important to use conditions as close as possible to those in which physiological oxidation occurs [17]. It should also be noted that, in general, antioxidant studies should be carried out at the temperature at which the antioxidant is required—for example, in food processing (frying), this temperature may be 160 °C or more. However, when conducted at 37 °C, LA takes a long time to oxidise, and times (i.e., end points of oxidation) ranging from 16 h to 12 days have been reported. For such long oxidations, high-throughput screening of antioxidants is not possible with the above assays. On the other hand, we have shown [18] that the TBARS assay (and possibly the FTC assay) has many of the features considered to be essential for measuring antioxidant activity [19], except the current lack of performing them as high-throughput assays.

The FTC and TBARS tests could potentially be accelerated by increasing the rate of oxidation. This can be performed by raising the temperature, use of a catalyst or use of an oxygen initiator such as transition metal ion in the presence of hydrogen peroxide. However, all these approaches may lead to a change in the mechanism of lipid oxidation [20], which may not give results for antioxidant activity that are valid compared to those at 37 °C. To assess antioxidant activity under accelerated conditions, validation parameters are needed, one of which would be order of antioxidant activity. As it is known that order of antioxidant activity can change under accelerated conditions [21], attempts to speed up the FTC or TBARS tests need to preserve the order established at 37 °C.

The objective of this study was therefore to develop a method, based on the FTC and TBARS tests, by altering various parameters, so that it becomes faster, and its validity can be checked through the order of antioxidant activity found at 37 °C. Such a method would provide complementary information to the well-established screening assays, such as ABTS, DPPH or ferric-reducing antioxidant power (FRAP), by revealing those antioxidants that show promise in inhibiting either primary (FTC assay) or secondary (TBARS assay) lipid oxidation products.

## 2. Materials and Methods 

### 2.1. Chemicals and Reagents

Linoleic acid (>99%) was purchased from Nu-Chek Prep Inc., Elysian, MIN, USA. (±)-6-Hydroxy-2,5,7,8-tetramethylchromane-2-carboxylic acid (Trolox) (97%), gallic acid (97.5%), (+)-catechin hydrate (>98%), (−)-epicatechin (>90%), caffeic acid (>98%), α-Tocopherol (>96%), quercetin (>95%), trichloroacetic acid (TCA) (>99%), thiobarbituric acid (TBA) (>98%), ammonium thiocyanate (>97.5%), polyoxyethylene sorbitan monolaurate (Tween 20) (97%) and iron(II) chloride (98%) were obtained from Sigma-Aldrich (Milwaukee, WI, USA). L-ascorbic acid (>99%) was purchased from Sigma-Aldrich (Shanghai, China), copper(II) chloride dihydrate (>99%), hydrogen peroxide (30%, v/v) and anhydrous sodium dihydrogen orthophosphate (sodium phosphate monobasic) (99%) from Chem-Supply (Adelaide, Australia), absolute ethanol (99.97%) from VWR Internationals (Fontenay-sous-Bois, France), methanol (99.99%) from Merck Chemicals (Darmstadt, Germany), nitric acid (70%, v/v) from Ajax FineChem Pty Ltd. (Auckland, New Zealand), hydrochloric acid (32%, v/v) from Ajax Finechem Pty Ltd. (Sydney, Australia), chloroform (>99%, HPLC grade) from Fisher Chemical (Loughborough, UK), cobalt(II) chloride hexahydrate (>98%) from M & B Laboratory Chemicals (Loughborough, UK) and anhydrous di-sodium hydrogen phosphate (sodium phosphate dibasic) (99.7%) from VWR International BVBA (Leuven, Belgium). Ultra-pure water (18 MΩ, Edwards Instruments Co, Sydney, Australia) was used throughout and glassware was soaked overnight in 10% (v/v) aqueous nitric acid (prepared from 70%, v/v, RCI, Labscan Ltd., Bangkok, Thailand). 

### 2.2. Experimental Design

In this study, an LA nano-emulsion was first prepared, and then was oxidised under a range of conditions: absence or presence of antioxidants; physiological temperature (37 °C) or accelerated conditions (50 and 60 °C); absence or presence of catalysts (Cu^2+^ or Co^2+^ and oxygen initiator (Fe^2+^ + hydrogen peroxide)). After oxidation, peroxides and TBARS were measured. All solutions, including LA emulsions, were freshly prepared before conducting the experiments. 

#### 2.2.1. Preparation of Solutions

##### Antioxidants

A range of antioxidants, including Trolox, gallic acid, ascorbic acid, quercetin, (+)-catechin, (–)-epicatechin, caffeic acid and α-tocopherol, were used. Quercetin and α-tocopherol were dissolved in methanol, while other antioxidants were dissolved in 50% (v/v) aqueous methanol. Trolox was used at the final concentrations of 25, 250 or 400 µM, and other antioxidants at 250 µM in oxidation samples consisting of a total 5 mL of LA emulsion, phosphate buffer and antioxidant solution. A final concentration of 250 µM of Trolox, gallic acid, ascorbic acid or quercetin was used for the experiments, involving added catalysts or added oxygen initiator. Aqueous methanol, 50% (v/v), without added antioxidant was used as control solvent.

##### Catalysts and Oxygen Initiator

Aqueous solutions (0.05 M) of copper(II) chloride and cobalt(II) chloride were prepared and 1 mM was used as the final concentration in the oxidation mixture [16,22]. For the oxygen initiator (Fe^2+^ + hydrogen peroxide), 3 µM iron(II) chloride and 2 µM hydrogen peroxide solutions were prepared separately, from which 0.04 µM Fe^2+^ and 0.01 µM hydrogen peroxide were used as the final concentrations [23].

##### Phosphate Buffer

A 0.2 M aqueous sodium phosphate buffer was prepared at a pH of 7. First, 0.2 M solutions of sodium phosphate monobasic (NaH_2_PO_4_) (solution A) and sodium phosphate dibasic (Na_2_HPO_4_) (solution B) were prepared in water. Then, 19.5 mL of A and 30.5 mL of B were mixed together in a volumetric flask, and the volume was brought to 100 mL with water. The resulting pH was adjusted to pH 7 (John Morris Scientific Pty Ltd., Sydney, Australia) by dropwise additions of solution A or B.

##### Linoleic Acid (LA) Nano-Emulsion (Oil-in-Water)

A 0.02 M LA (pH 7) nano-emulsion (droplet size < 250 nm in diameter, range 94–243 nm) was prepared in phosphate buffer following the method of Yen and Hsieh [8]. Briefly, 0.2804 g of LA and 0.2804 g Tween 20 were mixed in 50 mL 0.2 M (pH 7) phosphate and vortexed for 5 s. A milky mixture was produced, and the resulting pH was adjusted to pH 7, as above. The container of LA mixture was kept on ice until nano-emulsion preparation using the homogenisation procedure below.

##### Homogenisation and Nano-Emulsion Formation

Coarse pre-emulsions of LA were first prepared using an Ultra Turax homogeniser (T25 basic, Janka & Kunkel IKA Labortechnik, Selangor, Malaysia) at speed 4 (19,000 rpm) for 6 min. Nano-emulsions were then formed by passing the coarse mixture through a pneumatically driven high pressure valve homogeniser (Emulsiflex-C5, AVESTIN Inc., Ottowa, ON, Canada) three times at pressures between 70–140 × 10^3^ kPa. While collecting the emulsion, the container was also kept on ice to avoid oxidation. After homogenisation, a clear solution with no creaming or no separation of layers was obtained. Creaming was visually examined to see whether there was a change in turbidity between the top and bottom layers of the emulsion.

##### Droplet Size (Zeta-Average[d]) and Poly-Dispersity Index (PDI) Measurements

The mean droplet size (diameter) and poly-dispersity index (PDI) of the nano-emulsions (50 µL diluted in 4950 µL water, 25 °C) were measured in duplicate using a dynamic light scattering Zetasizer (Model ZEN 3600, Nano-ZS, Malvern, ATA Scientific, Malvern, UK) [24]. Droplet size and PDI were automatically calculated by the Zetasizer nano-ZS software (version 6.2). PDI is a dimensionless measure of the size distribution of oil droplets, and is calculated using equation σ^2^/Z_p_, where σ = standard deviation and Z_p_ = average diameter of droplets. A high value (maximum 1) shows a polydisperse system and a low value (minimum 0) denotes a monodisperse system. 

#### 2.2.2. Oxidation 

Three types of oxidation samples with or without antioxidants, catalysts and oxygen initiator were prepared in 2.5 mL LA emulsion (0.02 M pH 7) and 2 mL phosphate buffer (0.2 M pH 7) for oxidative studies at 37 °C or accelerated temperatures:

1. Antioxidant oxidation sample at 37, 50 or 60 °C: added 0.5 mL 25, 250 or 400 µM antioxidant/control solvent;
2. Antioxidant with added catalysts sample at 37 °C: 0.25 mL 250 µM antioxidant solution/control solvent + 0.1 mL catalyst solution + 0.15 mL methanol;
3. Antioxidant with added oxygen initiator sample at 37 °C: 0.25 mL 250 µM antioxidant solution/control solvent, 0.07 mL iron(II), 0.03 mL hydrogen peroxide solution and 0.15 mL methanol.

All solutions were mixed together in a screw-capped 50 mL centrifuge tube and vortexed for 5 s. The cap was then loosely attached to allow sufficient oxygen ingress and the mixture was oxidised in a water bath (±1 °C, Thermoline Scientific, Sydney, Australia). Sample 1 was oxidised between 10 to 96 h while samples 2–3 were oxidised for 10 h. Two 0.1 mL aliquots of the oxidation samples were taken at different intervals of oxidation, and peroxides and TBARS were measured using the procedures below.

#### 2.2.3. Measurement of Peroxides

Peroxides were measured following the ferric thiocyanate method by Yen and Hsieh [8]. Briefly, an aqueous solution of ethanol (4.7 mL, 75% (v/v)), aqueous ammonium thiocyanate (0.1 mL, 30% (w/v)), the above oxidation sample (0.1 mL) and aqueous iron(II) chloride (0.1 mL, 0.02 M in 3.2% (v/v) hydrochloric acid) were sequentially added and vortexed for 5 s. After exactly 3 min incubation, absorbance was measured at 500 nm against a blank containing all reagents except LA (Bio-Rad Smartspectro plus spectrophotometer (Sydney, Australia)). A control was performed with the LA sample but without the antioxidant. The antioxidant activity (% inhibition of peroxides formation) was calculated using Equation (1):

% inhibition of peroxides = [(A_0_ − A_s_)/A_0_] × 100, (1)

where A_0_ = absorbance in absence of antioxidant, A_s_ = absorbance in presence of antioxidant.

#### 2.2.4. Measurement of Thiobarbituric Acid Reactive Substances (TBARS)

TBARS were measured following the TBARS method by Stoilova [15]. Briefly, an aliquot of 0.1 mL oxidation sample was added to 2 mL TBA–TCA solution (20 mM TBA in 15% (w/v) aqueous TCA) in a 10 mL centrifuge tube and vortexed for 5 s. The mixture was heated in a water bath (100 °C) for 15 min and cooled to room temperature. Then, 2 mL chloroform was added and vortexed for 5 s. The mixture was centrifuged (Eppendorf Centrifuge 5810) at 2000 rpm for 15 min. The supernatant was carefully transferred to a plastic cuvette (10 mm) and absorbance was measured at 532 nm against a blank using all reagents except LA. Control sample preparation and measurement of antioxidant activity (% inhibition of TBARS formation) were performed as per the ferric thiocyanate method discussed above.

### 2.3. Statistical Analysis

All samples were prepared in triplicate (analytical replicates) and results are expressed as mean ± standard deviation. The statistical significance of differences was determined by one way ANOVA and post hoc least significant difference (LSD) using SPSS software, version 20 (SPSS Inc., Chicago, Ill, USA). Results were considered to be statistically significant at *p* < 0.05.

## 3. Results and Discussion

### 3.1. Preparation and Initial Testing of Linoleic Acid (LA) Nano-Emulsions

Oxidation of a lipid substrate is related to the size of the droplets in an emulsion [25] and to the polydispersity of droplet size (polydispersity index, PDI). Initially, two batches of LA (see below) were used to prepare two separate emulsions. The emulsion from batch I had a droplet size of 185.1 ± 0.2 nm, and a PDI of 0.3 ± 0.0, while the emulsion from batch II had a droplet size of 243 ± 0.4 nm, and a PDI 0.4 ± 0.0.

As mentioned above, one of the issues we reported [11] with using LA on water as an oxidation system within which to measure antioxidant activity, was the lack of reproducibility of results. Part of this variability was traced to different batches of LA giving different results. Therefore, a starting point to developing the method was to test two batches of LA in two separate emulsions.

**Figure 1:**
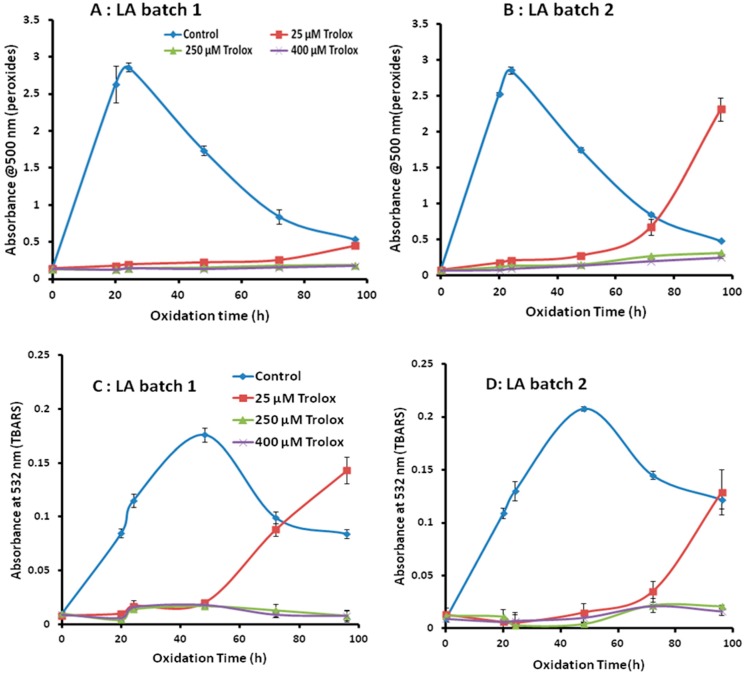
Absorbance values (*n* = 3) for peroxides (linoleic acid (LA) emulsion batch 1 (**A**) and LA emulsion batch 2 (**B**)) measured by ferric thiocyanate (FTC) assay, for TBARS (LA emulsion batch 1 (**C**) and LA emulsion batch 2 (**D**)) measured by thiobarbituric acid reactive substances (TBARS) assay (oxidation at 37 °C, error bars are based on standard deviation).

With these emulsions, oxidation was conducted at 37 °C for over 96 h, monitoring A_500_ values for peroxides and A_532_ for TBARS. Several aspects of reproducibility were evaluated, such as consistency of time to obtain maximum amount of peroxides and TBARS, intra-batch precision, and inter-batch variability in LA oxidation. In Figure 1A,B, maximum peroxide formation occurred at 24 h for both batches, with no statistically significant difference between the two A_500_ values (batch 1, 2.64 ± 0.25; batch 2, 2.85 ± 0.05; *p* > 0.05). For these two batches, both inter- and intra-batch precision (small error bars) were acceptable. In our previous study [11], there was some batch-to-batch variability in pre-formed peroxides, which seemed to lead to variability in peroxide measurement during oxidation; however, here, any pre-formed peroxides were either diluted or removed during the preparation of the emulsion (A_500_ values—0). Thus, formation of peroxides was consistent in the emulsion system, in contrast to the multi-phase system [11].

Maximum TBARS formation occurred at 48 h (A_532_ values—0.2 A for both batches) (Figure 1C,D). As expected, this is later than for peroxides, since TBARS are secondary oxidation products formed from the breakdown of peroxides. At 24 h reaction, there was no significant difference (*p* > 0.05) for the A_532_ values between batches (batch 1, 0.11 ± 0.01; batch 2, 0.13 ± 0.01) indicating minimal inter-batch variability in TBARS formation at this time point. However, after 48 h, differences in the amounts of TBARS formed between the batches became apparent. For example, at 72 h in batch I, the A_532_ value was 0.10, while in batch II the A_532_ value was 0.15. It is not clear what was driving this variability, but our previous work [11] suggested that peroxide breakdown may sometimes occur in an unpredictable way. Nevertheless, the goal of the study was to develop a rapid method of analysis and therefore what occurs later in the reaction will not impact on the validity of the results in the early stages.

Also apparent from Figure 1C,D is that the intra-batch variability was also low (small error bars on the plot). Therefore, oxidation of LA in an emulsion is much more consistent for TBARS measurement in terms of intra- and inter-batch variability, contrary to our previous study [11]. This is likely due to the better mixing of the various phases in an emulsion, compared with the three separate phases in the original study—aqueous, lipid and gas phase (air).

Having established good precision for the control system, it was also necessary to establish precision of the assays in the presence of an antioxidant. This is because it has been found, particularly for TBARS [9], that lower precision can occur when an antioxidant is present in the system. Furthermore, in our previous study [11], it was found that precision can vary depending on the concentration of antioxidant for both FTC and TBARS. Therefore in this work, three concentrations of Trolox were tested: 25, 250 and 400 µM. Figure 1A–D shows that the intra-batch precision for A_500_ and A_532_ values in the presence of all of the concentrations of Trolox was consistently high (small error bars). For batch-to-batch consistency, emulsions with 250 and 400 µM Trolox showed good reproducibility over 96 h. On the other hand, the emulsion with 25 µM Trolox showed variable formation of peroxides and TBARS after 48 h. As 25 µM Trolox is much less concentrated compared to 250 and 400 µM solutions, therefore later in the reaction (i.e., after 48 h) when the concentration of Trolox has decreased, various reactions may occur that lead to inconsistent amounts of peroxides and TBARS being formed. However, as noted above, this stage of the reaction is not of interest in the pursuit of a rapid antioxidant assay.

Although the oxidation of LA emulsion showed good intra- and inter-batch precision in the absence or presence of Trolox at 24 h (for maximum peroxides) and 48 h (for maximum TBARS), these times of oxidation are too long to be of use in antioxidant screening studies. Likewise, it is more convenient in a screening application to measure antioxidant activity at the same oxidation time, rather than two different times for two different tests. Therefore, as a starting point for further studies to reduce oxidation time (below), 20 h was chosen as the common time at which to measure peroxides and TBARS. As can be seen from Figure 1, 20 h gives good reproducibility, both in the control and with Trolox at all concentrations tested, in both the FTC and TBARS tests.

### 3.2. Method Optimisation

Increasing the temperature of reaction, addition of metal catalysts or addition of oxygen initiators may all be used to speed up the oxidation of LA, and hence reduce reaction times. However, all methods have the potential to alter the mechanism of oxidation, such that antioxidant activity measured under one set of conditions may not be the same as that measured at another. For example, Frankel [21] observed that ascorbic acid was less effective than ascorbyl palmitate at 45 °C in soybean oil, while it became more effective than ascorbyl palmitate, and even butylated hydroxyanisole (BHA) and butylated hydroxytoluene (BHT), at 98 °C. Therefore, to validate the method, some measure was needed to ensure that oxidation and antioxidant activity were comparable under accelerated conditions. To this end, we used the order of antioxidant activity, established at 37 °C after 20 h reaction, as the reference by which to evaluate subsequent reactions. Also, the time course of the formation of peroxides and TBARS was monitored to ensure that the progress of the reaction was consistent under different accelerated conditions.

#### 3.2.1. Oxidation at 37 °C

Trolox, ascorbic acid, gallic acid and quercetin were investigated to determine the order of antioxidant activity. Trolox was chosen as a reference as it was used during characterisation of the emulsion system in Section 3.1, above, and is often used to quantify antioxidant capacity (e.g., the TEAC parameter—Trolox equivalent antioxidant capacity [26]). Ascorbic acid, gallic acid and quercetin were chosen as they are commonly used in antioxidant activity studies as reference compounds [8,27] and are also commonly available in nature. Additionally, the antioxidants cover a range of polarities (log *K*_OW_ from –1.85, ascorbic acid to 1.48 for quercetin [28]). Two concentrations (25 and 250 µM) of the antioxidants were investigated. From these, a suitable concentration was selected to establish the order of antioxidant activity. Finding a suitable concentration is important, as at too high a concentration, all compounds may be equally effective as antioxidants (or may become prooxidant), while at too low a concentration, they might be all equally ineffective. Thus, a concentration is required that gives a good spread of activities.

**Figure 2:**
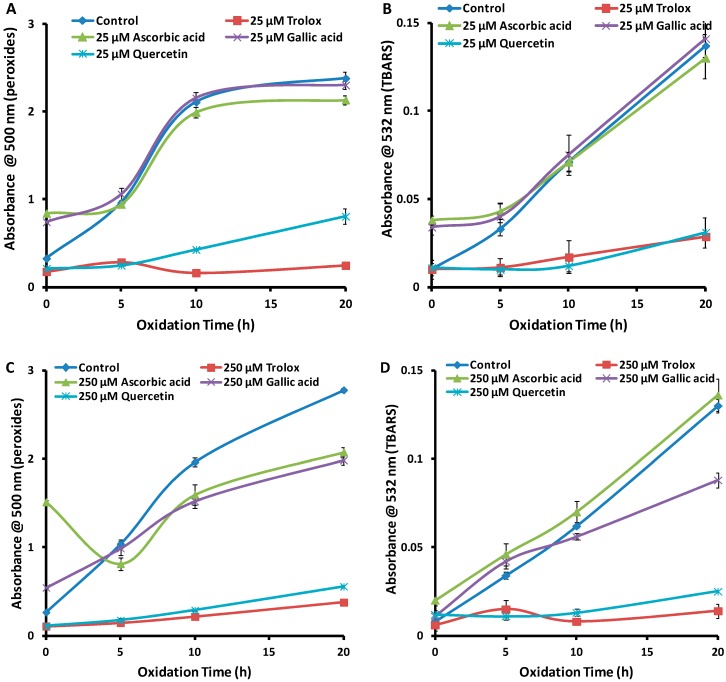
Absorbance values (*n* = 3) for (**A**) peroxides (FTC assay) and (**B**) TBARS at 25 µM, for (**C**) peroxides and (**D**) TBARS at 250 µM antioxidants (oxidation at 37 °C). Error bars are based on standard deviation.

In this part of the study, a total of nine emulsions (mean droplet size 110 ± 2 nm, and mean PDI 0.3 ± 0.03) were prepared using the above procedure. Figure 2A,B shows the time course oxidation of LA at 0, 5, 10 and 20 h in the absence or presence of 25 µM antioxidants. At 20 h oxidation, Trolox and quercetin showed antioxidant activity in both FTC and TBARS assays. On the other hand, gallic acid and ascorbic acid showed little or no antioxidant activity in comparison with the control. Indeed, at 0 h they appeared to be prooxidant. Alternatively, the non-zero absorbances at *t* = 0 h may be due to impurities in the antioxidants, reacting with the colourimetric reagents. Ultimately, these five *t* = 0 h points did not impact the overall conclusions of the study and were not investigated further. The main conclusion from Figure 2A,B is that half of the antioxidants tested were not effective at a concentration of 25 µM, and hence this was not a useful concentration for the optimisation of the method.

In contrast, 250 µM of all tested antioxidants at 20 h showed appreciable antioxidant activity in the FTC test (Figure 2C) and three antioxidants in the TBARS assay (Figure 2D), compared to the control. Only ascorbic acid showed no antioxidant activity in the TBARS assay. Thus, 250 µM was an effective concentration compared to 25 µM, and hence was chosen for further studies (below) to decrease the reaction times of these assays. It can be noted that the results presented here are consistent with previous studies in the literature—Trolox was reported to be more active than ascorbic acid [8] in an LA emulsion, while quercetin was reported to be more active than gallic acid in a camellia oil emulsion [29].

Figure 2C,D also shows that the order of antioxidant activity is the same for both the FTC and TBARS tests at 20 h. That is, Trolox > quercetin > gallic acid > ascorbic acid, and this order will be used to validate the results obtained below, under conditions of accelerated oxidation. Since this order of activity was also evident after 10 h, 10 h was used as the endpoint of the reaction in subsequent studies.

#### 3.2.2. Oxidation at Accelerated Conditions

**Figure 3:**
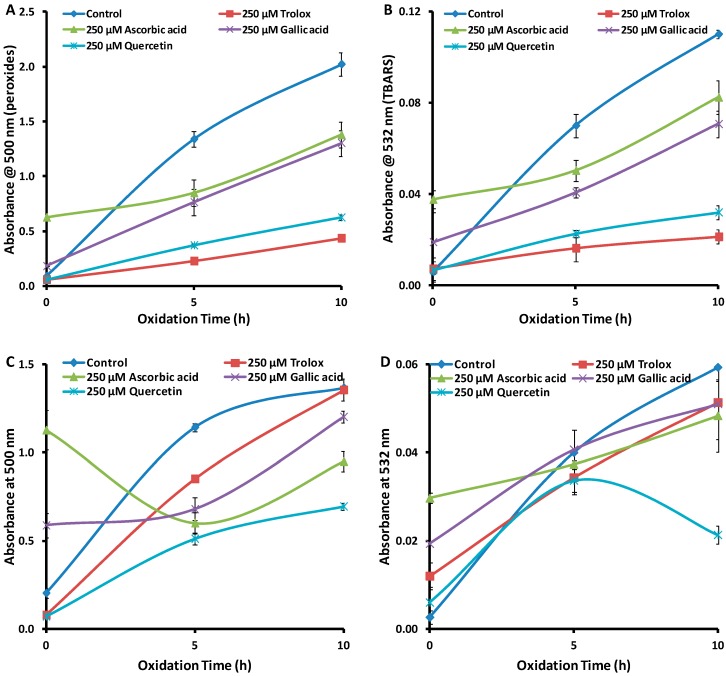
Absorbance values (*n* = 3) for (**A**) peroxides (FTC assay) and (**B**) TBARS at 50 °C, for (**C**) peroxides and (**D**) TBARS at 60 °C and 250 µM antioxidants. Error bars are based on standard deviation.

As a 10 °C increase in temperature can double the oxidation rate [30], performing the oxidation at 50 °C or 60 °C may be able to reduce reaction time by up to a factor of four. Thus, an antioxidant assay based on these tests could be performed in as little as 5 hours (one-quarter of 20 h, cf. 37 °C), providing the mechanism does not change at higher temperatures. Figure 3A–D shows the time course of peroxide and TBARS formation at these elevated temperatures up to 10 h. In the control at 50 °C, the rate of formation of TBARS (Figure 3B) increased relative to the rate at 37 °C (Figure 2B), but not by a factor of two. There was no apparent increase in the rate of formation of peroxides (Figure 3A, cf. Figure 2A), and this may be due to the fact that the rate of peroxide decomposition had also increased at the higher temperature. Further increase in temperature to 60 °C actually led to a decrease in the amount of peroxides relative to both lower temperatures (37 °C and 50 °C). For TBARS, the amount formed at 10 h at 60 °C was the same as that formed at 37 °C. The results at 60 °C suggest that the breakdown of peroxides had been accelerated, as the A_500_ value was less than at the lower temperatures. Furthermore, the breakdown of peroxides did not form TBARS—the A_532_ value at 60 °C was less than at 50 °C and no greater than at 37 °C. Therefore, on the basis of the results of the control, it would appear as though the mechanism of oxidation at 60 °C changed from that at 37 °C.

In the presence of antioxidants, the production of both peroxides (Figure 3A) and TBARS (Figure 3B) were inhibited at 50 °C. Figure 3A shows that for peroxides, oxidation at 5 h and 10 h at 50 °C showed the same order of antioxidant activity—Trolox > quercetin > gallic acid ≈ ascorbic acid. Similarly, in Figure 3B, for TBARS, the same order of antioxidant activity was observed at 5 h and 10 h. The order of antioxidant activity for both peroxide and TBARS formation at 50 °C was the same as that observed at 37 °C, indicating that the results for the assay were valid at the higher temperature according to the validation parameter established above.

On the other hand, Figure 3C shows that at 60 °C, the time course of oxidation reactions and the order of antioxidant activity were changed compared to the results at 37 °C and 50 °C, above. Over the time course of the reaction, all antioxidants showed some activity, especially at 5 h. However, Trolox, which had been the most effective antioxidant under all previous conditions, had no antioxidant activity at 10 h reaction. In contrast, quercetin, which had also showed strong antioxidant activity under previous conditions, was still quite active at 60 °C. More complex behaviour was observed for TBARS formation in the presence of antioxidants (Figure 3D). At 5 h, no compounds showed significant antioxidant activity, and at 10 h, only quercetin inhibited TBARS formation. The lack of congruence of the behaviour of the antioxidants at 60 °C with that at 37 °C is further evidence that the mechanism of oxidation has altered at the higher temperature.

On the basis of the effect of temperature in accelerating oxidation, it is evident that the order of antioxidant activity and the time course of oxidation can only be preserved at 50 °C for 5 and 10 h, compared to 37 °C and 20 h oxidation. Thus, 5 h oxidation at 50 °C can be reliably used in an assay for the measurement of antioxidant activity. Under these conditions, the assay time is one quarter that at 37 °C, offering the possibility of an assay that could be used in preliminary screening.

To investigate the effect of catalytic metal ions, Cu^2+^ and Co^2+^ ions were selected to study their potential to accelerate LA oxidation in the emulsion system. Copper(II) ions were chosen because of their reported strong prooxidant effect [16], and also since they are present in biological systems, while Co^2+^ ions have been reported to be powerful oxidising agents [31].

**Figure 4:**
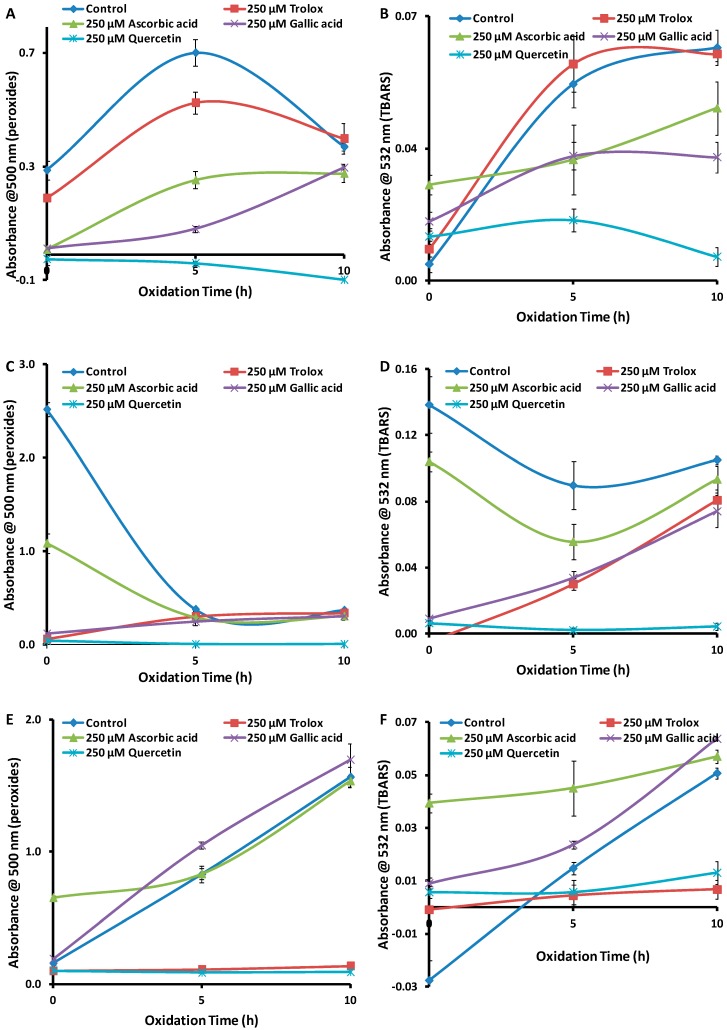
Absorbance values (*n* = 3) of (**A**) peroxides (FTC assay) and (**B**) TBARS for 1 mM Cu^2+^, (**C**) peroxides and (**D**) TBARS for 1 mM Co^2+^, (**E**) peroxides and (**F**) TBARS for (0.04 µM Fe^2+^ + 0.01 µM H_2_O_2_) at 37 °C and 250 µM antioxidants. Error bars are based on standard deviation.

It is immediately apparent from Figure 4A–D that none of the time course curves match those in Figure 2C,D for peroxide or TBARS formation at 37 °C. Thus, it can be concluded that the addition of the metal ions altered the oxidation mechanism, relative to the validation parameters established above. Therefore, the addition of metal ions is not a valid means to increase the reaction rate, with a view to decreasing reaction time, to make these assays suitable for high-throughput screening.

Although not directly related to the goal of this study, some comments on the effects of the metal ions, as demonstrated in Figure 4A–D, are warranted. Firstly, the formation of peroxides is affected by the presence of the metal ions, but not in a consistent way. Figure 4A (control) shows that, in the presence of Cu^2+^ ions, the maximum peroxide formation occurred at 5 h, with A_500_ peaking at 0.70. This contrasts with the system with no metal ions (Figure 1) where the maximum absorbance of 3.0, was reached after 24 h. The shorter time to peak absorbance suggests the Cu^2+^ ions did in fact increase the rate of formation of peroxides, but the lower value for the maximum absorbance suggests that they also increased the rate of peroxide degradation [32].

In contrast, in the presence of Co^2+^ ions (Figure 4C control), peroxides were at a maximum (2.50 A) on day 0, and decreased rapidly over 5 h. Here, it would appear that Co^2+^ ions caused immediate formation of peroxides and possibly catalysed their degradation [33], i.e., degradation was essentially complete in 5 h, but in the absence of Co^2+^ ions (Figure 1A control), degradation took > 60 h.

Dissimilar behaviour in peroxide formation was also observed when antioxidants were added to the systems with different metal ions. Figure 4A shows the order of antioxidant activity was quercetin > gallic acid > ascorbic acid > Trolox in the samples with Cu^2+^ ions after 5 h, but after 10 h, only quercetin continued to show antioxidant behaviour. In the samples with Co^2+^ ions (Figure 4C), there was no antioxidant behaviour observed for any antioxidants, other than quercetin, at 5 h or 10 h. These results are difficult to interpret, but it is of note that quercetin was effective against peroxide formation in the presence of both metal ions, while Trolox was almost ineffective in preventing peroxide formation in the presence of Cu^2+^, in sharp contrast to its behaviour under previous conditions (Figure 1A,B, Figure 2C and Figure 3A). This result may confirm observations by others that Trolox can become a pro oxidant in the presence of copper ions [22].

The results for the TBARS test (Figure 4B,D) show that addition of Cu^2+^ or Co^2+^ to the emulsion leads to oxidation behaviour that is not consistent with the validation conditions. Again, both the time-course of the formation of oxidation products and the order of antioxidant activity have been affected by the presence of the metal ions. Thus, it may be concluded that addition of these metal ions is not a valid way to decrease oxidation time to develop a high-throughput assay.

Dutta and Maharia [23] used Fe^2+^ + H_2_O_2_ in an LA emulsion to accelerate oxidation, while measuring antioxidant activity of plant extracts in the TBARS test. Figure 4E,F shows the results of the time course of oxidation using the same oxygen initiator. In the control sample at 10 h, peroxide formation (A_500_ = 1.6) was slightly less than under validation conditions (Figure 2A, A_500_ = 2.1), possibly due to the presence of metal ions catalysing the breakdown of the peroxides (*vide infra*). TBARS formation at 10 h (Figure 4F, A_532_ = 0.05) was also lower than under validation conditions (Figure 2B, A_532_ = 0.07). As discussed above, the breakdown of peroxides does not necessarily generate TBARS, and this appears to be the case here.

In this system, only Trolox and quercetin showed antioxidant activity against formation of peroxides (Figure 4E) and TBARS (Figure 4F). Neither gallic acid nor ascorbic acid were able to inhibit formation of peroxides or TBARS in contrast to the validation conditions (Figure 2C,D). Therefore, it may be concluded that accelerating oxidation with Fe^2+^ + H_2_O_2_ is not valid for decreasing reaction times.

On the basis of these overall effects, some accelerated oxidation parameters—the raise of temperature to 60 °C, the use of Cu^2+^ and Co^2+^ catalysts and use of an oxygen initiator—can be excluded for optimising faster FTC and TBARS assays, as these parameters cannot provide the time course for oxidation, nor the order of antioxidant activity, as established at 37 °C for 20 h. On the other hand, the order of antioxidant activity and time course of oxidation reactions were preserved at both 5 and 10 h for 50 °C. Thus, 5 h reaction for 50 °C was exclusively selected as the valid accelerated oxidation time and temperature.

### 3.3. Method Performance

To further assess the utility of the accelerated antioxidant tests, several different antioxidants were trialed. Trolox was used as a reference, and (+)-catechin, (−)-epicatechin, caffeic acid and α-tocopherol were selected as belonging to different chemical classes with different polarities, as well as representing some antioxidants commonly found in plants [34].

**Figure 5:**
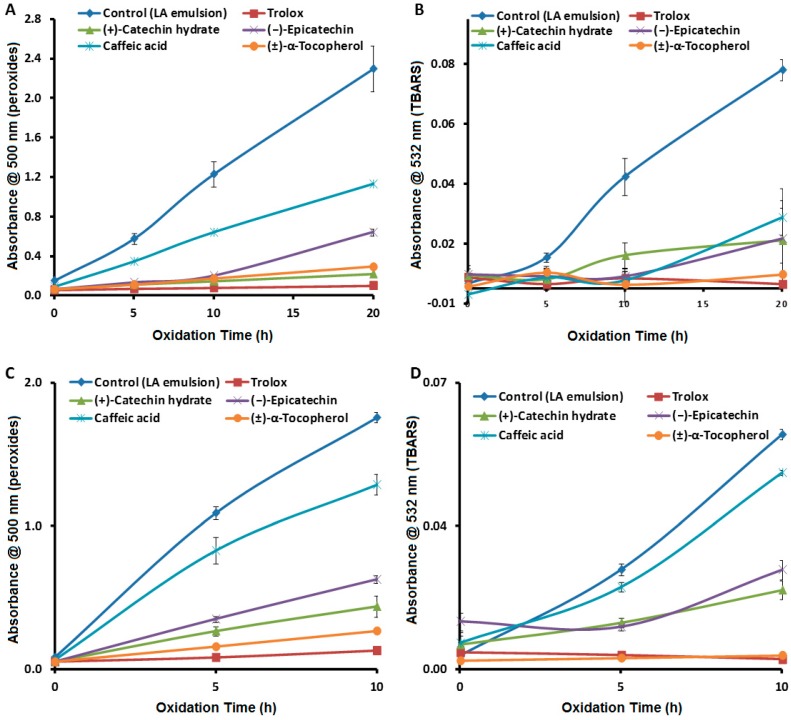
Absorbance values (*n* = 3) for (**A**) peroxides (FTC assay) and (**B**) TBARS at 37 °C, for (**C**) peroxides (FTC) and (**D**) TBARS at 50 °C and 250 µM antioxidants. Error bars are based on standard deviation.

Figure 5A,B shows the time course for generation of peroxides and TBARS, respectively, for the oxidation of an LA emulsion at 37 °C in the presence of the above antioxidants. For peroxides (Figure 5A), the order of activity at 20 h was Trolox ≈ (+)-catechin ≈ α-tocopherol > (−)-epicatechin > caffeic acid. For TBARS (Figure 5B), the order of antioxidant activity at 20 h was Trolox ≈ α-tocopherol > (+)-catechin ≈ (−)-epicatechin ≈ caffeic acid. Under accelerated oxidation conditions at 50 °C (Figure 5C,D), the order of antioxidant activity at 5 h was consistent with that at 37 °C and 20 h. The consistency in the order of antioxidant activity between the two sets of reaction conditions with these antioxidants suggests that performing the FTC and TBARS assays under the accelerated conditions is a valid way to reliably assess antioxidant activity, but in a much shorter period of time. Therefore, the method can be used reliably for high-throughput measurement of antioxidant activity of new compounds or plant extracts.

## 4. Conclusions

Previously, the TBARS test has been shown to be highly suited to use in antioxidant studies [18], with the main drawback being the long times involved in oxidising a substrate. In this study, we successfully accelerated the oxidation of LA through heating the reaction to 50 °C, measuring peroxides as primary oxidation products and TBARS as secondary oxidation products. At this temperature, the time course of oxidation with and without various antioxidants, and order of antioxidant activity, were shown to be similar to those when oxidation was carried out at 37 °C. On the other hand, heating to higher temperature (60 °C), the use of metal ion catalysts (Cu^2+^, Co^2+^) or an oxygen initiator (Fe^2+^ + H_2_O_2_), led to different time courses of oxidation and/or different orders of antioxidant activity. Therefore, none of these latter methods are suitable for accelerating oxidation in antioxidant tests using a LA emulsion.

When heating a LA emulsion at 50 °C, the order of antioxidant activity was established in 5 h, which is considerably less time than reported in other studies. Therefore, a method has been developed that has the potential to be very useful in, for example, screening studies of bioactives in traditional medicinal plants. Ideally, the method could be refined further to provide quantitative data, rather than the qualitative data on order of activity. Even so, the current method provides complementary information to the more widely used assays (Folin-Ciocalteu, ferric ion reducing antioxidant power DPPH, ABTS, etc.), which are undertaken without a lipid substrate. With this method, a follow up study is now underway, examining the antioxidant properties of Australian native medicinal plants.
